# 3,5-Diiodo-l-Thyronine Increases Glucose Consumption in Cardiomyoblasts Without Affecting the Contractile Performance in Rat Heart

**DOI:** 10.3389/fendo.2018.00282

**Published:** 2018-05-30

**Authors:** Ginevra Sacripanti, Nhat Minh Nguyen, Leonardo Lorenzini, Sabina Frascarelli, Alessandro Saba, Riccardo Zucchi, Sandra Ghelardoni

**Affiliations:** Dipartimento di Patologia Chirurgica, Medica, Molecolare e dell’Area Critica, University of Pisa, Pisa, Italy

**Keywords:** T2, heart perfusion, cardiomyoblasts, glucose consumption, T2 uptake

## Abstract

3,5-diiodo-l-thyronine (T2) is an endogenous derivative of thyroid hormone that has been suggested to regulate energy expenditure, resting metabolic rate and oxygen consumption with a mechanism that involves the activation of mitochondrial function. In this study, we focused on the cardiac effects of T2, which have been poorly investigated so far, by using both *in vitro* and *ex vivo* models. As a comparison, the response to T3 and T4 was also determined. Rat cardiomyoblasts (H9c2 cells) were used to determine T2, T3, and T4 uptake by high-performance liquid chromatography–tandem mass spectrometry. In the same experimental model, MTT test, crystal violet staining, and glucose consumption were investigated, using T2 concentrations ranging from 0.1 to 10 µM. To assess cardiac functional effects, isolated working rat hearts were perfused with T2, T3, or T4 in Krebs-Ringer buffer, and the hemodynamic variables were recorded. T2 was taken up by cardiomyoblasts, and in cell lysate T2 levels increased slowly over time, reaching higher concentrations than in the incubation medium. T2 significantly decreased MTT staining at 0.5–10 µM concentration (*P* < 0.05). Crystal violet staining confirmed a reduction of cell viability only upon treatment with 10 µM T2, while equimolar T3 and T4 did not share this effect. Glucose consumption was also significantly affected as indicated by glucose uptake being increased by 24 or 35% in cells exposed to 0.1 or 1.0 µM T2 (*P* < 0.05 in both cases). On the contrary, T3 did not affect glucose consumption which, in turn, was significantly reduced by 1 and 10 µM T4 (−24 and −41% vs control, respectively, *P* < 0.05 and *P* < 0.01). In the isolated perfused rat heart, 10 µM T2 produced a slight and transient reduction in cardiac output, while T3 and T4 did not produce any hemodynamic effect. Our findings indicate that T2 is taken up by cardiomyoblasts, and at 0.1–1.0 µM concentration it can modulate cardiac energy metabolism by increasing glucose consumption. Some evidence of toxicity and a transient impairment of contractile performance are observed only at 10 µM concentration. These effects appear to be specific for T2, since they are not reproduced by T3 or T4.

## Introduction

3,5-Diiodo-l-thyronine (T2), an endogenous metabolite of thyroid hormones (TH), has been described as a peripheral mediator of several TH metabolic effects (1). Although conversion of 3,5,3′-triiodothyronine (T3) to T2 has not yet been demonstrated *in vitro*, indirect evidence indicates that T2 is, indeed, formed from T3 *in vivo*, through deiodination (2). Both T2 and T3 increase resting metabolic rate, but the response to T2 shows a more rapid onset (3). These findings have induced to hypothesize that T2 might mediate some of the short-term effects of TH and that it might be involved, like T3, in physiological processes leading to increased energy expenditure (3). While the response to T3 was principally mediated by nuclear receptors, the effects of T2 were independent of protein synthesis (4) suggesting that they were probably due to a direct interaction with mitochondria.

Several reports have shown that, in rats, acute or chronic T2 administration causes significant changes in mitochondrial activities, stimulating fatty acid oxidation, and decreasing hepatic lipid accumulation (5–9). At the dosage 0.25 µg/g body weight for 4 weeks i.p. (10), T2 prevented body weight gain in rats fed with a high-fat diet, without inducing T3-related undesirable side effects, namely tachycardia, cardiac hypertrophy, and decreased TSH levels. In this models, T2 stimulated mitochondrial uncoupling, decreased ATP synthesis, and increased hepatic fatty acid oxidation rate, thus counteracting obesity. Additionally, T2 induced biochemical and structural shifts toward glycolytic myofibers and prevented an increase in serum triglycerides and cholesterol (10, 11). On the whole, it has been clearly established that T2 exerts hepatic antilipidemic effects, but its physiological relevance in skeletal and cardiac muscle is still unclear. In skeletal muscle, Moreno et al. (12) reported increased insulin sensitivity upon T2 administration, particularly increased protein kinase B phosphorylation, and sarcolemmal GLUT4 accumulation. In another investigation, T2 induced proton leak in skeletal muscle mitochondria obtained from hypothyroid rats (13).

Chronic administration of a low T2 dosage to two healthy volunteers increased resting metabolic rate and decreased body weight, by inducing a reduction in steatosis and in the total serum cholesterol levels, without affecting cardiac function (14). Conversely, a recent study reported that, at a higher dosage (2.5 µg/g), T2 administration exerted thyromimetic effects, since it induced cardiac hypertrophy and an overall genomic effect similar to that produced by the TH (T3) (15). However, the cardiac effects of T2 have not been extensively investigated so far. Therefore, in this study we explored the functional, metabolic, and toxic effects of T2 using both *in vitro* and *ex vivo* models of cardiac preparations.

## Materials and Methods

### Chemicals

H9c2 (2–1) rat cardiomyoblast cell lines were obtained from American Type Culture Collection (Manassas, VA, USA). [^3^H]-ryanodine was obtained from New England Nuclear (Milan, Italy). Ryanodine was purchased from Abcam, UK. Unless otherwise specified, all reagents were obtained from Sigma-Aldrich (St. Louis, MO, USA). Solvents for HPLC–MS/MS measurements were HPLC-grade, and the other chemicals were reagent-grade.

### Cell Culture and Treatment

Rat cardiomyoblasts (cell line H9c2) were cultured in DMEM supplemented with 10% (vol/vol) FBS, 1 mM pyruvate, 100 U/ml penicillin, and 100 µg/ml streptomycin at 37°C in a humidified atmosphere containing 5% CO_2_ and subcultured before confluence. To assess glucose uptake, H9c2 were seeded in six-well plate (5 × 10^5^ cells/well), grown to 80% of confluence with standard medium and washed twice with PBS before treatment. Then, cells were exposed for 4 h to exogenous T2 (0.1–10 µM) in 1 ml of the same DMEM base (phenol free) supplemented with 0.7 mg/ml glucose. Control cells were incubated with DMEM containing the same volume of vehicle. Cell culture medium was then collected and glucose concentration was evaluated in medium with a spectrophotometric assay kit (Sigma-Aldrich). Hexokinase activity was assessed in cell lysate with a colorimetric assay kit (BioVision, Milpitas, CA, USA). Enzyme activity was evaluated following kit protocol. The absorbance was read at 450 nm. Metabolite concentrations were referred to the total protein content of whole-cell lysates (16).

### Uptake of T2 and HPLC–MS/MS Assay Technique

The experiments aimed at evaluating hormone uptake were performed as previously described, with minor modifications (17). Briefly, cells (H9c2) were seeded into 24-well plate (8.5 × 10^4^ cells/well) and grown to 80% confluence. At the start of each experiment, the culture medium was removed, and after washing with PBS, fresh medium containing 100 nM T2, 50 nM T3 or T4 was added. The plate was returned to a humidified atmosphere of 5% CO_2_ at 37°C, and the medium was removed from each well at specific time points and frozen at −80°C until extraction. At the end of the experiment, cell plates were washed with PBS and frozen. Cell lysis was carried out using 100 µl of 0.1 M NaOH and samples were neutralized by adding 10 µl of 1 M HCl. After pH neutralization, 390 µl MeOH were added and samples collected and centrifuged for 10 min at 14,000 × *g*, and the supernatant was evaporated under N_2_ at 40°C. Dry samples were then reconstituted using 50 µl of water/acetonitrile (70:30 by volume) containing 0.1% formic acid, and analyzed using HPLC coupled tandem mass spectroscopy, as elsewhere described (17). Cell culture medium was extracted using a liquid–liquid method: 1 ml of methyl tert-butyl ether was added and then the mixture was vigorously shaken for 30 s. Then the mixture was briefly spinned at 14,000 × *g* to completely separate the organic and aqueous phases. The organic phase was then collected and the extraction process was repeated twice. At the end of the procedure, the collected organic phases were evaporated under N_2_ at 40°C. Dry samples were then reconstituted using 50 µl of water/acetonitrile (70:30 by volume) containing 0.1% formic acid and analyzed using HPLC coupled tandem mass spectroscopy assay, as detailed below. The positive ion mode method included: ionspray voltage, 5.00 kV; gas source 1, 70; gas source 2, 55; turbo temperature, 650°C; entrance potential, 10 V; collision-activated dissociation gas pressure, 12 mPa. HPLC runs were based on the following mobile-phase gradient: solvent A, methanol/acetonitrile 1:4, containing 0.1% formic acid; solvent B, water containing 0.1% formic acid. The gradient started at 5% A increasing to 65% at 8.5 min, then to 100% at 9 min, maintained until 11 min, with subsequent re-equilibration at 5% for further 2.5 min. The flow rate was 0.4 ml/min.

The selected reaction monitoring (SRM) method transitions and the related parameters for T2 were set as follows. Transitions are: 525.9→352.9, 525.9→381.8, 525.9→479.9 KDa; declustering potential 87.0 kV; collision energy 40.8, 27.6, and 26.0 kV, respectively; collision exit potential 10.3,11.2, and 14.2 kV, respectively. The SRM method transitions for the detection of T3 and T4 were set as described previously (17). Medium peak area at time zero was taken as reference area for the other time points.

### Cell Viability Test

Cell viability was assessed by using two different assays: the 3-(4,5-dimethylthiazol-2-yl) 2,5-diphenyltetrazolium bromide (MTT) test (18) and crystal violet staining (19). Cells were seeded in 96-well microtiter plate at a density of 5,000–10,000. Twenty four hours after, T2, T3, or T4 were added at different concentrations and cell viability was determined 24 h after incubation. MTT (0.5 mg/ml) was added to the medium, and after an additional 4 h incubation, SDS–HCl (0.05 mg/ml) was added to solubilize formazan salt. After 18 h, the absorbance of the solution was read at 570 nm in a microplate reader (BioRad Laboratories, Italy). Since T2 is known to affect mitochondrial activity and MTT test is based on mitochondrial function, crystal violet staining was performed according to Feoktistova et al. (19) with minor modifications. Upon iodothyronine treatment, cells were washed gently with warmed PBS, then stained 10 min at room temperature with crystal violet solution (0.2% crystal violet in 2% ethanol). The plate was washed twice with deionized water, and then a 1% SDS solution was added to each well, and the plate was agitated until complete solubilization of the staining that was read at 570 nm.

### Isolated Heart Perfusion and Ryanodine Binding

Experimental procedures were approved by the ethical committee of the University of Pisa (protocol no. 51814/2016). The investigation conforms with the Guide for the Care and Use of Laboratory Animals published by the US National Institutes of Health (NIH publication no. 85-23, revised 1996).

Male Wistar rats (275–300 g body weight), fed a standard diet, were anesthetized with a mixture of ether and air. The heart was then quickly excised and perfused according to the working heart technique, as described previously (17). The perfusion buffer included (mM): NaCl, 118; NaHCO_3_, 25; KCl, 4.5; KH_2_PO_4_, 1.2; MgSO_4_, 1.2; CaCl_2_, 1.5; glucose, 11. Perfusions were carried out using 200 ml of recirculating buffer, which was equilibrated with a mixture of O_2_ (95%) and CO_2_ (5%). Temperature was kept between 36.8 and 37°C, and the pH was 7.4. Powerlab/200 (ADInstruments, Castle Hill, Australia) was used for hemodynamic variable acquisition. The height of the atrial chamber was set at 20 cm, corresponding to a filling pressure of 15 mmHg. During the experiment, after an equilibration period of 10 min, T2, T3, or T4 (0.1–10 µM) was added to the perfusion buffer and hearts were perfused for another 50 min. In the control group, hearts were perfused only with standard buffer and vehicle. At the end of the perfusion, hearts treated with or without 10 µM T2 were homogenized in five volumes of 300 mM sucrose and 10 mM imidazole (pH 7.0 at 4°C) and high affinity ryanodine binding was determined on the crude homogenate (20). Briefly, vesicles were incubated at 37°C in a buffer containing 25 mM imidazole (pH 7.4 at 37°C), 1 M KCl, 0.4–40 nM [3H]ryanodine (6 Ci/mmol), 0.950 mM EGTA, and 1.013 mM CaCl_2_ (free Ca^2+^ concentration was 20 µM). After 60 min, the binding reaction was stopped by filtration through cellulose nitrate filters which were washed twice with 25 mM imidazole and 1 M KCl (washing buffer). Radioactivity was counted at 60% efficiency in TRI-CARB 2800 TR Liquid Scintillation Analyzer (Perkin Elmer, Italy). Incubations were performed in duplicate and nonspecific binding was measured in the presence of 10 µM unlabeled ryanodine. Saturation experiments were analyzed by nonlinear fitting of a single binding site model.

### Statistical Analysis

Results are expressed as the mean ± SEM. Differences between groups were analyzed by one-way or two-way ANOVA as detailed for each figure. In the experiments aimed at determining differences vs a single control group, Dunnett’s *post hoc* test was applied. When the experimental setting included only two groups, statistical differences were determined by unpaired *t*-test. The threshold of statistical significance was set at *P* < 0.05. GraphPad Prism version 6.0 for Windows (GraphPad Software, San Diego, CA, USA) was used for data processing and statistical analysis.

## Results

### Cellular Uptake of Iodothyronines

**Figure 1:**
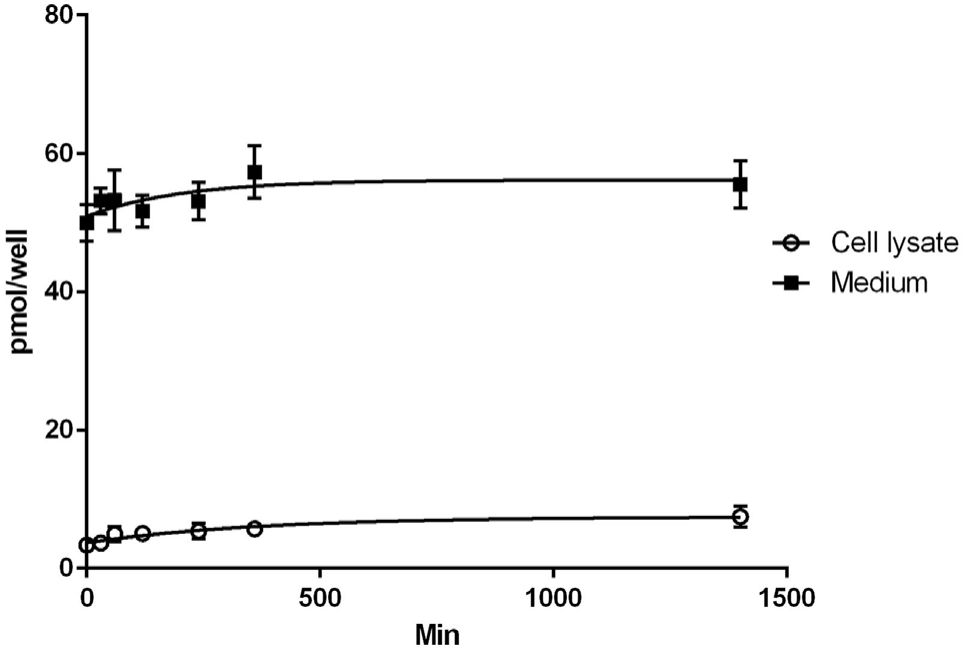
Uptake of T2. Results of T2 uptake in H9c2 cell during 24 h of incubation with 100 nM T2 in medium. Assays were performed at different times in the incubation medium and in the cell lysate. Values are mean ± SEM of results derived from three uptake experiments.

The results of the T2 uptake experiments in H9c2 cells are shown in Figure 1. Uptake was carried out with medium (0.5 ml) containing 100 nM T2 (50 pmol/well). In lysate, T2 concentration increased over time reaching a value of about 14 nM (7 pmol/well) after 24 h, while in medium steady state concentration was reached after 6 h, and it averaged about 110 nM (55 pmol/well). So the overall recovery of T2, after 24 h infusion, was slightly higher than 100%. Since lysate volume was in the order of 0.02–0.03 ml, the actual cellular concentration at the end of the treatment can be estimated to be about 250–350 nM. From preliminary experiments, similar results were obtained in the presence of 50 nM T3 or T4, whose cellular uptake after 24 h was on the order of 10 and 2% of the medium concentration, respectively (data not shown).

### Cell Viability

**Figure 2:**
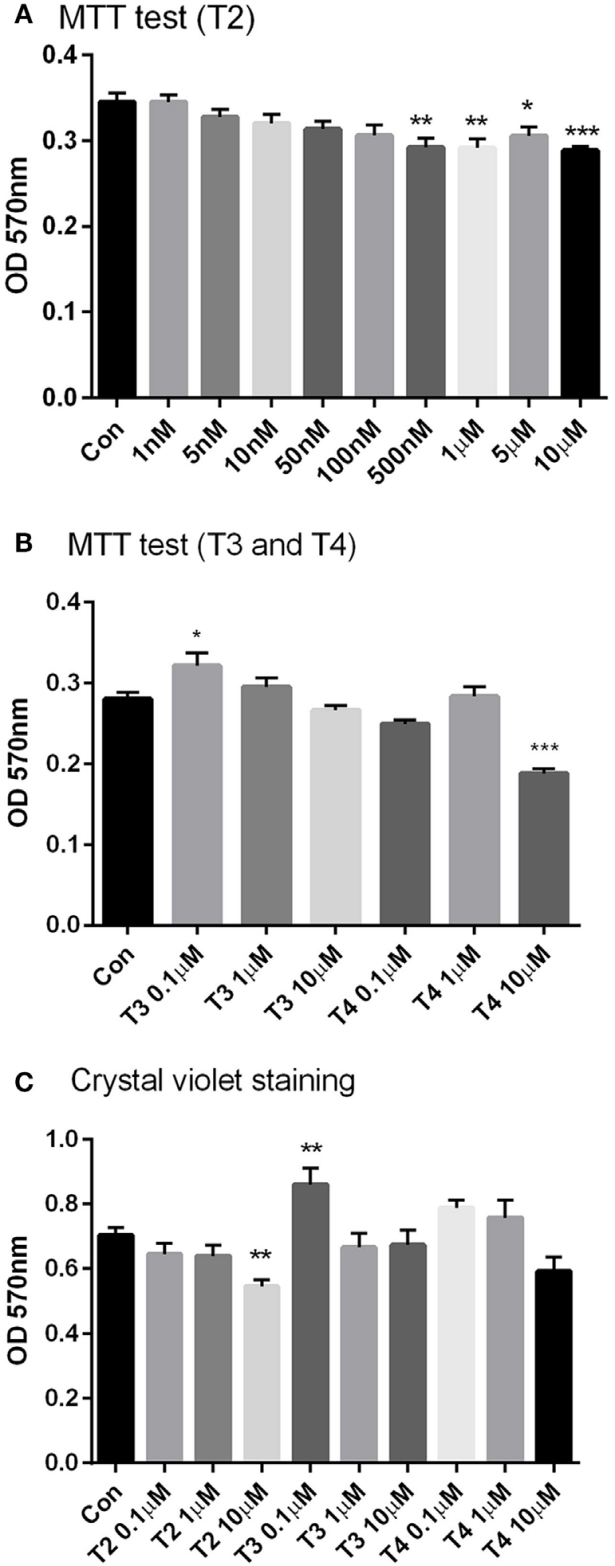
Cell viability. H9c2 cells were incubated for 24 h with a wide range of T2 (1 nM–10 μM), T3, and T4 (0.1–10 µM) concentrations in medium incubation and then the cell viability tests, MTT test **(A,B)**, and crystal violet assay **(C)** were performed. All treatments received the same amount of vehicle. Control group was incubated with medium containing the same volume of vehicle. Data are plotted as means of 4–6 replicas ± SEM [one-way ANOVA, *P* < 0.0001, Dunnett’s *post hoc* test for multiple comparison, *P < 0.05, **P < 0.01, ***P < 0.001 vs control (con), *n* = 4–6 per group].

After 24 h incubation with T2, MTT assay was carried out in H9c2 cell lines. As shown in Figure 2A, incubation with T2 at concentrations ≥500 nM caused 15–16% decrease in MTT staining, compared to vehicle (*P* < 0.05 by one-way ANOVA test and Dunnett’s *post hoc*). This may imply decreased cell viability (and/or reduced oxidative metabolism). To get a better insight, we also performed crystal violet staining. Crystal violet binds to DNA and proteins of cells that did not lose their adherence, and is considered as an alternative index of viability. As shown in Figure 2C, crystal violet staining was reduced (*P* < 0.05) only at the highest T2 concentration (10 µM), whereas it was not affected by equimolar T3 or T4 and it was actually increased in the presence of 0.1 µM T3 (*P* < 0.05 vs control). Similar results were obtained in MTT staining by T3 (*P* < 0.05 0.1 µM vs control), while 10 µM T4 significantly reduced cell viability by 33% (*P* < 0.001 vs control, Figure 2B).

### Glucose Consumption and Hexokinase Activity

**Figure 3:**
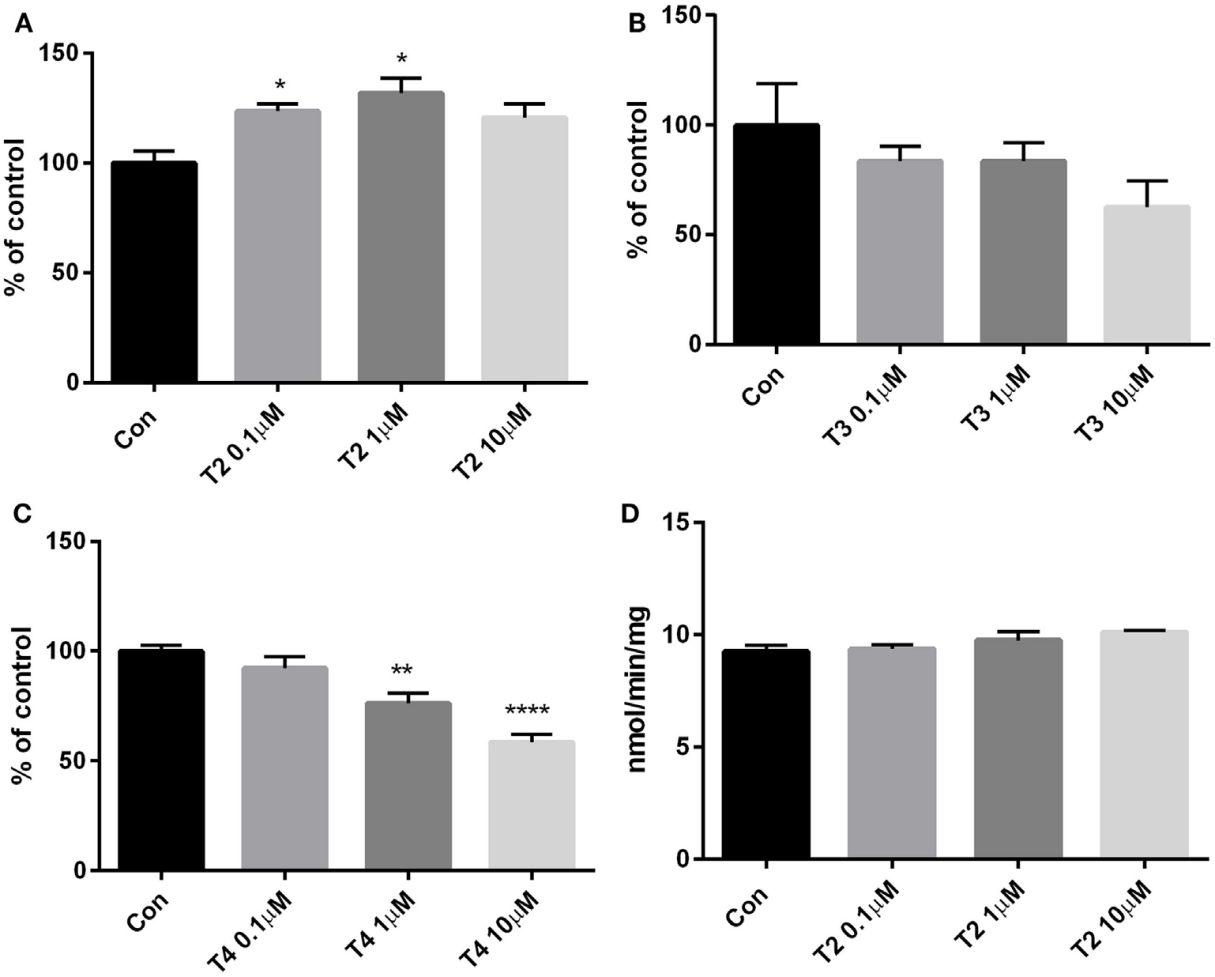
Glucose consumption and hexokinase activity. Glucose concentration was assayed in cell medium after 4 h treatment with **(A)** T2, **(B)** T3, or **(C)** T4 (0.1–10 µM). Results are the difference between the initial glucose concentration in medium (0.707 mg/ml, assessed with the glucose assay kit) and the final concentration, normalized to the total content of protein in lysate. Control cells were incubated with medium containing the same volume of vehicle. **(D)** Hexokinase activity assay was performed in cell lysates upon treatment with T2. Values are mean ± SEM of 3–4 replicates and are expressed as percent of control in the glucose consumption or as NADH nmol/min/mg in the enzyme activity. [one-way ANOVA, *P* < 0.05, Dunnett’s *post hoc* test for multiple comparison, *P < 0.05, **P < 0.01 vs control (con), *n* = 3–4 per groups].

To assess glucose consumption, H9c2 cells were incubated for 4 h in phenol red-free DMEM containing 0.7 g/l glucose, as described in Section “Materials and Methods.” At the end of treatment, glucose concentration was assayed in the medium and the results were expressed as the difference between the initial and final concentrations, and normalized to the total protein content of cell lysates. As shown in Figure 3A, T2 caused a 23–30% increase in glucose consumption if administered at the concentrations of 0.1 or 1 µM (*P* < 0.05 vs control by one-way ANOVA test and Dunnett’s test), by a mechanism which does not involve hexokinase, as revealed by enzymatic activity being unaffected by T2 (Figure 3D). At 10 μM T2 concentration, glucose consumption was also enhanced, but the difference vs control did not reach statistical significance (0.066 ± 0.003 vs 0.055 ± 0.003 mg glucose/mg protein, *P* = NS). By comparison, glucose consumption was not affected by 0.1–10 µM T3 (Figure 3B), while it was significantly reduced in cell exposed to 1–10 µM T4, by 24 and 41%, respectively (*P* < 0.05 and *P* < 0.001, respectively; see Figure 3C).

### Heart Perfusion and Ryanodine Binding

**Figure 4:**
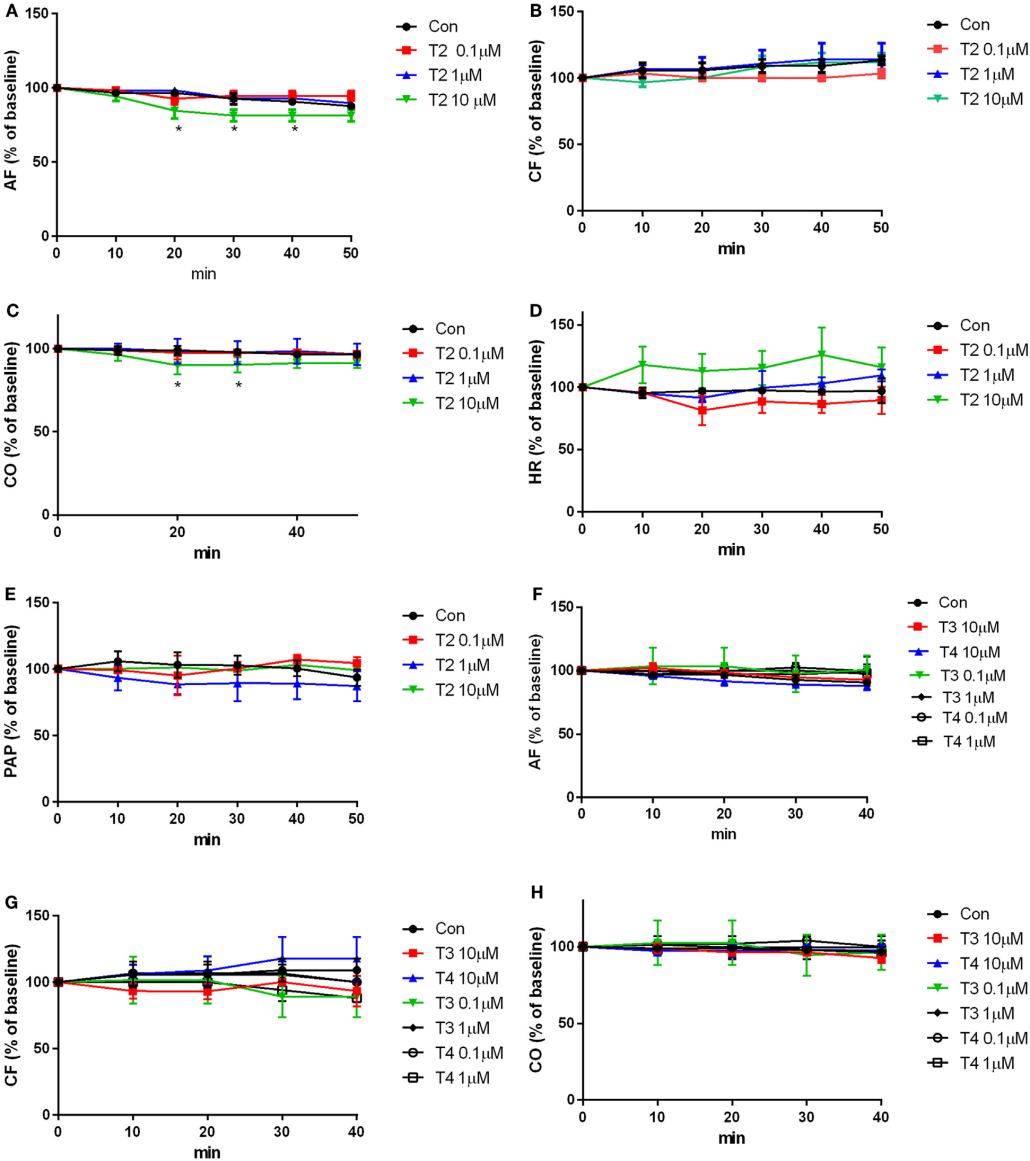
Contractile performance. Heart was perfused for 50 min with buffer containing the vehicle or iodothyronines (0.1–10 µM). Hemodynamic variables were measured every 10 min of perfusion and are expressed as percentage of the basal values, that were measured after 10 min of perfusion, i.e., before any addition to the perfusion buffer supplemented with **(A–E)** T2, **(F–H)** T3, **(F–H)** T4, or vehicle. Results represent mean ± SEM of three hearts per group. [*P < 0.05 for the effect of treatment, by two-way ANOVA, Dunnett’s *post hoc* test for multiple comparison, *P < 0.05 vs control (con), *n* = 3 per groups]. Abbreviations: AF, aortic flow; CF, coronary flow; CO, cardiac output; HR, heart rate; PAP, peak systolic aortic pressure.

The results of the perfusion experiments are shown in Figure 4. Baseline values of the hemodynamic variables averaged as follows: aortic flow (AF) 37.3 ± 0.6 ml/min, coronary flow (CF) 19.3 ± 0.6 ml/min, cardiac output (CO) 56.7 ± 0.7 ml/min, heart rate (HR) 215.6 ± 9.8 beats/min, and peak systolic aortic pressure (PAP) 182.8 ± 6.8 mmHg. T2 (0.1-10 µM) was added 10 min after the beginning of the perfusion, and hearts were perfused for another 50 min with T2, while in the control group the same amount of vehicle was added. After 20 min of perfusion with 10 µM T2, a slight but significant reduction was observed in CO and in AF (AF: −10–12% at 20–40 min *P* < 0.05; CO: 9–10% at 20–30 min *P* < 0.05; see Figures 4A,C). The other hemodynamic variables, namely the CF, HR, and PAP were not affected by T2 perfusion (Figures 4B,D,E). As a comparison, perfusions with 0.1, 1, and 10 µM T3 or T4 did not produce any significant changes in contractile performance (Figures 4F–H, AF, CF, and CO).

**Figure 5:**
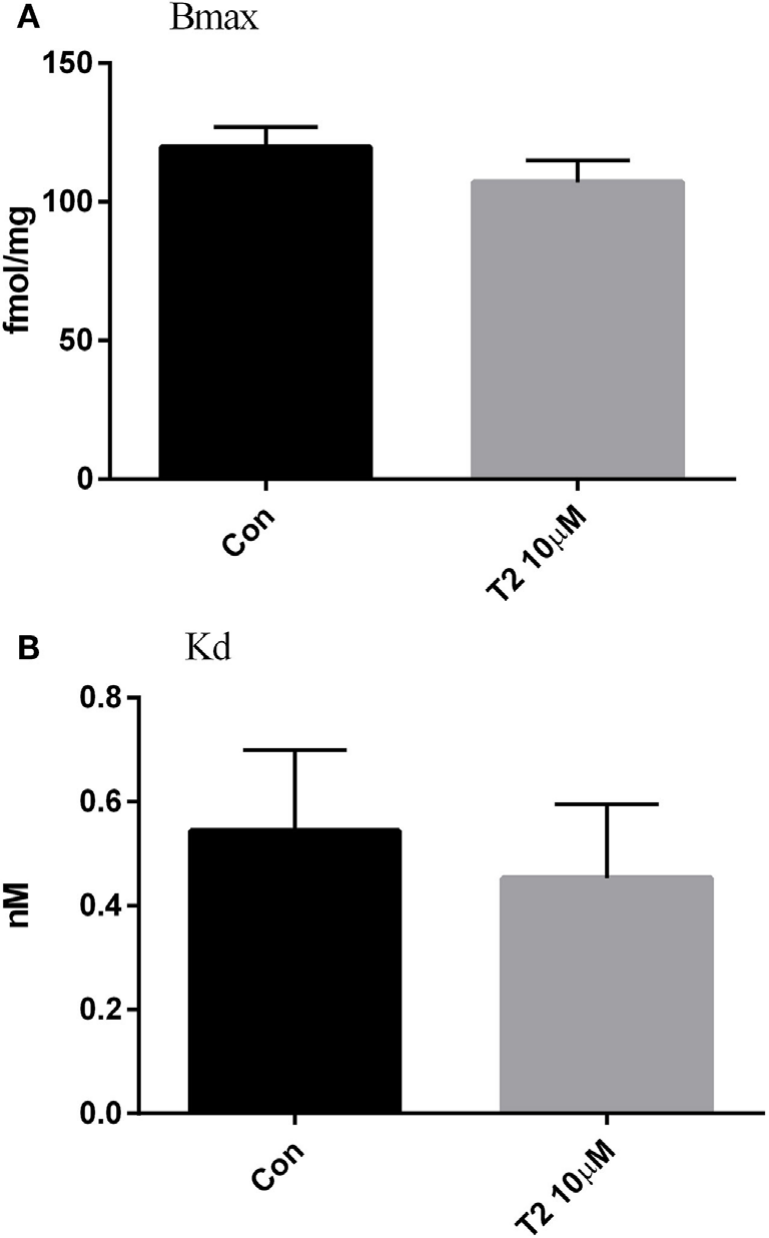
Ryanodine binding. Effect of T2 on the density of ryanodine binding sites [Bmax, fmol/mg, **(A)**] and affinity for ryanodine [Kd, nM, **(B)**] in crude cardiac homogenates. Ryanodine binding was determined in ventricle homogenate after 50 min of perfusion. Histograms represent mean ± SEM derived from three hearts per group. Unpaired *t*-test yielded *P* = ns for the difference between groups.

In ryanodine binding experiments, crude homogenates of hearts perfused with 10 µM T2 were analyzed. We explored only the concentration that affected, albeit only transiently, the CO. As shown in Figure 5, T2 did not modify ryanodine binding: either the number of binding sites (Figure 5A) or the affinity for ryanodine (Figure 5B) were unchanged by T2 perfusion (Bmax averaged 107.0 ± 7.9 vs 119.7 ± 7.3 fmol/mg; Kd averaged 0.45 ± 0.14 vs 0.54 ± 0.16 nM, *P* = NS in all cases).

## Discussion

T2 is a putative derivative of the TH whose effects on lipid metabolism are well established. On the contrary, the cardiac actions of T2 are less known and still controversial. In the present work, we analyzed the effects of T2 on cardiac tissue in perfused rat hearts and H9c2 cell cultures, using equimolar dosages of T3 and T4 as a control.

We observed that T2 can be taken up and accumulated in cardiomyoblasts. T2 was rapidly absorbed, since its presence was detected in cell lysate after a few minutes of incubation, with a complete recovery after 24 h, suggesting that no T2 catabolites were produced under these experimental conditions, although additional investigations are needed to verify this hypothesis. At the end of the treatment, cell lysate concentration was estimated to average about 250–350 nM, exceeding the medium concentration by over 2–3-fold. Either MCT8 or MCT10 might be responsible for T2 uptake, since these transporters are relatively nonspecific for iodothyronines (21). However the molecular identity of T2 transporters is still unknown.

The absence of significant T2 catabolism is consistent with the fact that rat cardiomyocytes, and in general rodent hearts, express low concentration of type II deiodinase, which has higher affinity for T3 and T4 than type I deiodinase (22). Although type III deiodinases might be more abundant in cadiomyoblasts, considering their fetal origin and capacity of proliferation (type III deiodinase expression is correlated to proliferative activity and pluripotency) (22), their effect appeared to be negligible. We cannot exclude that T2 catabolism might be balanced by local production, since H9c2 cell line have been reported to produce low levels of TH in an appropriate environment (23).

The glucose consumption results showed that T2 increased cardiomyocyte glucose uptake, as already demonstrated in skeletal muscle by Moreno et al. (12), who have described an increase in myofiber glycolytic activity. Consistently, T2 has been reported to be able to increase GLUT4 concentrations on plasma membrane, by increasing AKT phosphorylation by insulin (12). This might suggest that the higher glucose uptake was a consequence of an increase in glycolytic flow, even though the hexokinase activity was not influenced by T2, as revealed by enzymatic assay. The latter appears to be a primary metabolic effect, since the hemodynamic variables were not modified, and the energy need for contraction was, therefore, putatively unchanged, as confirmed also by the absence of changes in sarcoplasmic reticulum calcium channels.

As to the underlying molecular mechanism, the relatively short time course of our experiments (1–4 h) does not suggest the occurrence of genomic effects. This is consistent with several previous studies [reviewed by Moreno et al. (24)], and with the observation that T2 has a low affinity for nuclear TH receptors (25), and in any case it is relatively selective for TR beta, while TR alpha is the predominant cardiac isoform (26, 27). On the whole, it is likely that T2 activates short-term mechanisms, acting directly on specific protein targets, possibly located in mitochondria (28).

*In vitro* viability tests indicated that T2 produced only minor toxic effects upon a chronic (24 h) treatment. MTT staining was slightly but significantly reduced at concentrations exceeding 500 nM. Since the MTT test depends on mitochondrial function, we also used the crystal violet assay, finding out some evidence of T2 toxicity only at the highest concentration (10 µM). As reported (19), crystal violet is a stain for viable adherent cells, able to bind to proteins and DNA. Cells undergoing cell death lose their adherence and are subsequently lost from the population of cells, reducing the amount of staining in cell culture. So, the decreased MTT staining observed at lower concentrations may be related to a reduced oxidative metabolism due to changes in mitochondrial function. On the other hand, the toxic effect was not reproduced by equimolar T3 and T4, and interestingly 10 µM T2, but not equimolar T3 or T4, produced a slight and temporary reduction of the CO in the isolated working rat heart model.

Our experimental findings are consistent with the clinical results reported by Antonelli et al. (14) upon a 4-week treatment (300 µg/day) in healthy volunteers: T2 decreased body weight, and enhanced resting metabolic rate without affecting cardiac contractility. On the contrary, Jonas et al. (15) observed that T2 induced cardiac hypertrophy in mice after 2 weeks of treatment at 2.5 µg/g.

Differences in T2 concentration are a crucial issue, potentially able to account for these discrepancies. The T2 dosages used in previous studies (28–31) were widely different, namely 0.1–100 nM (29), 10 nM (28), 0.01–10 µM (31), or 1–10 µM (30). Different serum levels have also been measured: depending on the assay method applied to quantify T2, its circulating concentration ranged from 20–50 to 400–500 pM (32–38). Higher serum concentrations (50–400 nM) were measured by Jonas et al. (15) in mice treated for 14 days with 0.25–2.5 µg/g T2. Interestingly10 μM T2, the highest dosage reported in *in vitro* studies (30, 31) was the only one for which a slightly cytotoxicity was observed in our cell line.

In conclusion, our findings show that T2 can increase cardiac glucose consumption even though the contractile performance was unchanged. In our model, only minimal toxic effects were observed, at concentrations substantially higher than those associated with metabolic effects. So far, T2 serum concentration has not been accurately assessed, and further investigations will be needed to develop, standardize, and validate appropriate analytical methods. Once endogenous T2 levels are determined, it will be possible to investigate its potential physiological, pathophysiological, or pharmacological relevance for the heart and other tissues.

## Data Availability Statement

Datasets are available on request. The raw data supporting the conclusions of the manuscript will be made available by the authors, without undue reservation, to any qualified researcher.

## Ethics Statement

Experimental procedures were approved by the ethical committee of the University of Pisa (protocol no. 51814/2016, for organ explants). The investigation conforms with the Guide for the Care and Use of Laboratory Animals published by the US National Institutes of Health (NIH publication no. 85-23, revised 1996).

## Author Contributions

GS, MN, and LL designed and carried out cell culture experiments. SF performed the *ex vivo* experiments on heart. AS carried out mass spectrometry measurements. RZ revised the manuscript and performed statistical analysis. SG designed and supervised the experimental work and wrote the manuscript.

## Conflict of Interest Statement

The authors declare that the research was conducted in the absence of any commercial or financial relationships that could be construed a potential conflict of interests.
